# Publishing 2D materials research towards industrialisation

**DOI:** 10.1038/s41467-022-29527-7

**Published:** 2022-04-13

## Abstract

Graphene and related two-dimensional (2D) materials have been at the core of intense research and development for over fifteen years, however the market penetration of products based on this technology has lagged behind expectations. At Nature Communications we wish to support research providing insights into the path towards the industrialisation of 2D materials. We introduce a Collection that encapsulates the recent progress and outstanding challenges faced by research on atomically thin materials, and we focus, in particular, on the potential of 2D technologies for future impact at the commercial level.

In the vast area of nanotechnology, a balance has long persisted between addressing fundamental questions, and pursuing system development and optimisation to enable commercial products. As ideas progressively mature, an increased effort is devoted to make research impactful by seamlessly linking it to an application.

Journals like *Nature Communications* are ideally placed to serve as a bridge between academia and industry, by hosting and catalysing applied nano-research at the forefront of industrial innovation. One criteria we consider when assessing such application-driven research is its technology readiness level (TRL). TRLs are nine indicators that measure the degree of technological maturity; an increase in TLR signals a progressive shift from the observation of the underlying mechanism to a fully functional demonstration of a complex system in its operational environment. For technologies based on 2D materials, a high TRL may, for example, underpin the integration of miniaturised electronic components in high-density chips for high-performance computing or internet-of-things.

While we remain interested in basic science, *Nature Communications* also aims to publish research on 2D materials that bridges the gap between the widely available TRL-4 demonstrations—that is, 2D technologies assessed and validated in academic laboratories—and more sophisticated prototype systems closer to commercial deployment.

While we remain interested in basic science, Nature Communications aims to publish research on 2D materials that bridges the gap between the widely available TRL-4 demonstrations—that is, technology assessed and validated in academic laboratories—and more sophisticated prototype systems ready for commercial deployment.

We appreciate that research that is high on the TRL scale may not necessarily offer a considerable degree of conceptual novelty, as the ground-breaking investigation of the operating principles may have already been reported. However, it is thanks to steady and careful device optimisation—along with the development of feasible strategies for performance enhancement—that technology gets to make a tangible societal impact. We feel that such applied research has a strong potential for supporting the UN *Sustainable Development Goals*, and we remain committed to covering it consistently in our pages.

The editors handling research on 2D technology at *Nature Communications* firmly believe in the importance of reproducibility, reliability, and benchmarking, particularly for device demonstrations with high TRL. These aspects are crucial to determine the extent to which laboratory prototypes are compatible with industrial environment standards. To aid the issue of reproducibility, we look for the use of quantitative methods, including statistical analyses on representative sets of devices (in addition to measuring the so-called hero device that features the best performance). We also welcome the adoption of standardised methodologies, accredited by international institutes like the International Electrotechnical Committee (IEC) and the International Organization for Standardization (ISO), for accurate nomenclature and characterisation of 2D materials. Our approach is in line with the recent guidelines of the *European Commission* aimed at mitigating the lack of reproducibility in scientific research.

To illustrate the potential of 2D technologies to climb the TRL ladder and gradually enter the marketplace, we have put together a Collection of studies (including original research articles, Reviews, Perspectives, Comments) published in *Nature Communications* highlighting recent advances in this diverse field. The selected works include applications at various TRL stages, and span nano-electronics, biosensing, memories, energy storage, robotics, nano-photonics, nano-medicine.

Graphene and related 2D materials have remained an active field of research in science and engineering for over fifteen years. So, where are the enabled products? To understand why the transition from laboratories to fabrication plants appear to lag behind expectations, we have commissioned a *Comment* where Lemme *et al*. summarise the main challenges and opportunities that have thus far prevented the commercialisation of these materials. According to the authors what is needed, but not yet available, are turnkey manufacturing solutions that would bring 2D materials into silicon semiconductor factories.

The existing challenges are also strongly dependent on the industrial-scale manufacturing of 2D films and powders of appropriate morphology and quality. In their *Comment* Choi et al. discuss the main state-of-the-art mass production techniques, their limitations, and opportunities for future improvement.

The importance of statistical analyses on 2D electronic devices and circuits is emphasised by Lanza *et al*. in a *Comment*, where the authors outline viable strategies resulting in research papers that are useful for the industry. Furthermore, the relevant performance metrics of 2D devices need to be reliably and transparently characterised, as explained by Prof Gerasimos Konstantatos in a *Comment* on the technological prospects of photodetectors based on atomically thin materials.

Although several important steps are still underway in order to reach high TRLs, researchers are working intensely to overcome the current limitations in performance and scalability of 2D materials. A strong collaboration between academic laboratories and industrial facilities is crucial to achieve these goals, and *Nature Communications* commits to offer a platform to promote this dialogue.

